# Do try this at home: Age prediction from sleep and meditation with large-scale low-cost mobile EEG

**DOI:** 10.1162/imag_a_00189

**Published:** 2024-06-21

**Authors:** Hubert Banville, Maurice Abou Jaoude, Sean U.N. Wood, Chris Aimone, Sebastian C. Holst, Alexandre Gramfort, Denis-Alexander Engemann

**Affiliations:** Université Paris-Saclay, Inria, CEA, Palaiseau, France; InteraXon Inc., Toronto, Canada; Roche Pharma Research and Early Development, Neuroscience and Rare Diseases, Roche Innovation Center Basel, F. Hoffmann–La Roche Ltd., Basel, Switzerland; Max Planck Institute for Human Cognitive and Brain Sciences, Department of Neurology, Leipzig, Germany

**Keywords:** brain age, electroencephalography, cognitive health, machine learning, biomarker discovery

## Abstract

Electroencephalography (EEG) is an established method for quantifying large-scale neuronal dynamics which enables diverse real-world biomedical applications, including brain-computer interfaces, epilepsy monitoring, and sleep staging. Advances in sensor technology have freed EEG from traditional laboratory settings, making low-cost ambulatory or at-home assessments of brain function possible. While ecologically valid brain assessments are becoming more practical, the impact of their reduced spatial resolution and susceptibility to noise remain to be investigated. This study set out to explore the potential of at-home EEG assessments for biomarker discovery using the brain age framework and four-channel consumer EEG data. We analyzed recordings from more than 5200 human subjects (18–81 years) during meditation and sleep, to predict age at the time of recording. With cross-validated$R^{2}$scores between0.3-0.5, prediction performance was within the range of results obtained by recent benchmarks focused on laboratory-grade EEG. While age prediction was successful from both meditation and sleep recordings, the latter led to higher performance. Analysis by sleep stage uncovered that N2-N3 stages contained most of the signal. When combined, EEG features extracted from all sleep stages gave the best performance, suggesting that the entire night of sleep contains valuable age-related information. Furthermore, model comparisons suggested that information was spread out across electrodes and frequencies, supporting the use of multivariate modeling approaches. Thanks to our unique dataset of longitudinal repeat sessions spanning 153 to 529 days from eight subjects, we finally evaluated the variability of EEG-based age predictions, showing that they reflect both trait- and state-like information. Overall, our results demonstrate that state-of-the-art machine-learning approaches based on age prediction can be readily applied to real-world EEG recordings obtained during at-home sleep and meditation practice.

## 1 Introduction

In recent years, the emergence of low-cost mobile electroencephalography (EEG) devices has made it possible to monitor and record brain activity in entirely new environments, dramatically improving access to the technology for various applications (Johnson & Picard, 2020;Krigolson et al., 2017;Niso et al., 2023). Wearable EEG devices typically have few channels, are wireless, and use dry electrodes but are significantly more affordable than their clinical counterparts. This makes them perfect candidates for translating EEG screening procedures to ecologically valid settings, for example, at home or wherever clinical or research infrastructure is not available. The accessibility and affordability of low-cost mobile EEG devices can open the door to the tracking of brain activity and health on a day-to-day basis. Still, a major obstacle to this goal is the need for large volumes of labeled clinical EEG data to develop pathology-specific predictive models that can deliver actionable biomarkers, that is, measurable characteristics of the brain that provide information about its function and pathology (Ward, 2010).

To address this challenge, it is worthwhile to explore applying pretext tasks to learn representations (Banville et al., 2021) or proxy measures (Dadi et al., 2021) in the context of wearable EEG. These techniques can distill biomedical information of interest in the scarcity or absence of direct labeled data and are related to transfer learning (Pan & Yang, 2010). In human neuroscience, predicting age from brain data has emerged as a popular blend of these concepts with the potential to deliver biomarkers of brain health and pathology by capturing deviations from what “normal” brains “look like” (Cole & Franke, 2017;Cole et al., 2018;Denissen et al., 2022). In this framework, the*brain age delta*Δ is defined as the difference between an age estimate (i.e., the age predicted by a model trained on a healthy population) and chronological age. A positive Δ can reflect premature aging or pathology that make affected brains “look” older by comparison, for example, caused by Alzheimer’s disease or disturbed sleep (Chu et al., 2023;Cole & Franke, 2017;Cole et al., 2018;Franke & Gaser, 2012;Lee et al., 2022;Weihs et al., 2021). Evidence on the clinical utility of brain age keeps accumulating. Most of the research involved has focused on structural magnetic resonance imaging (MRI), thus restricting brain age measurement to clinical and research settings, potentially neglecting opportunities to explore this technique in at-home and ambulatory settings outside MRI scanners, and severely limiting the feasibility of obtaining subject-wise repeated measures.

A recent line of research has provided first hints that the information obtained through functional neuroimaging modalities could provide similar, or even complementary, information on brain age (Engemann et al., 2020;Liem et al., 2017;Xifra-Porxas et al., 2021). Specifically, EEG-based brain age modeling with EEG has been explored during resting state (Al Zoubi et al., 2018;Vandenbosch et al., 2019), clinical screening (Sabbagh et al., 2020,^2023^), and sleep (Brink-Kjaer et al., 2020;Paixao et al., 2020;Sun et al., 2019;Ye et al., 2020). In light of these findings, low-cost mobile EEG (Hashemi et al., 2016) emerges as a promising tool for estimating brain age out-of-the-lab as part of a regular at-home screening procedure.

In this work, we analyzed consumer-grade EEG recordings from more than 5200 individuals to explore the feasibility of a real-world brain age metric. As clinical or cognitive-testing information was absent in the present study, our work focuses on establishing a methodology and benchmarks for brain age prediction from at-home EEG recordings. Our main contributions are as follows: (1) we present first age prediction results from large-scale real-world mobile EEG collected during meditation and sleep in a personal environment, (2) we evaluate the predictive power of different covariance-based EEG representations and provide insights into which types of features are useful for predicting age in this real-world context, and (3) we provide insights into the variability of age prediction results for longitudinal recordings spanning up to 529 days.

## 2 Methods

In this section, we describe how we trained machine-learning models to predict age from low-cost mobile EEG and how the resulting brain age metric was evaluated.

### 2.1 Machine-learning problem setting

We train a machine-learning model$f_{\Theta }$with parametersΘto predict the age$y^{\left(i\right)}$of a subjectigiven their EEG${\text{X}}^{\left(i\right)}\in {\mathbb{R}}^{C\times T}$, withCthe number of EEG channels andTthe number of time points. To do so,$f_{\Theta }$is trained to minimize the error between the true target age$y^{\left(i\right)}$and the predicted age${\hat{y}}^{\left(i\right)}$over a training set ofNsubjects, that is,i∈[1,N].

Multiple machine-learning pipelines have been proposed in the age regression literature, including “shallow” regressors fed with handcrafted features (e.g., summary statistics and power spectrum-derived descriptors) (Al Zoubi et al., 2018;Engemann et al., 2020;Sun et al., 2019) or with filterbank covariance-based features (Sabbagh et al., 2019,^2020^,^2023^), as well as deep neural networks fed with minimally preprocessed data (Brink-Kjaer et al., 2020;Gemein et al., 2020;Roy et al., 2019). Here, following the results of a recent extensive benchmark of brain age prediction approaches (Engemann et al., 2022), we use vectorized filterbank covariance matrices as features$\nu \in {\mathbb{R}}^{F}$and a Ridge regression model (Hoerl & Kennard, 1970) as$f_{\Theta }$. This pipeline yielded competitive age prediction performance with deep learning models on four M/EEG datasets inEngemann et al. (2022). It is also easily interpretable (as shown inSection 3.2), whereas interpreting deep neural networks applied to raw data remains challenging (Roy et al., 2019), and it can be trained efficiently, which facilitates estimating variability in prediction performance. Finally, the use of covariance matrices as features allows disentangling correlations between electrodes and systematically increases the expressiveness of mathematical representations of the EEG in relation to the underlying data-generating process, for example, when a nonlinear relationship between EEG source powers and the target of interest (i.e., subject age in this work) can be expected.

We next describe the datasets and the EEG preprocessing methodology, before presenting our machine-learning pipeline in more detail (Section 2.4).

### 2.2 Datasets

We used real-world mobile EEG recordings collected by anonymized users of the Muse S headband (RRID:SCR_014418; InteraXon Inc., Toronto, Canada). These data were collected in accordance with the privacy policy (July 2020) users agree to when using the Muse headband^1^and which ensured their informed consent concerning the use of EEG data for scientific research purposes. The Muse S is a wireless, dry EEG device (TP9, Fp1, Fp2, TP10, referenced to Fpz) with a sampling rate of 256 Hz. Two types of recordings were used in this study: (1) meditation recordings with neurofeedback and (2) overnight sleep recordings. In both cases, recordings were carried out by users through the*Muse*application on iOS or Android mobile devices.^2^In meditation recordings, users performed an eyes-closed focused attention task with real-time auditory feedback. This feedback was driven by the users’ EEG using a proprietary trade-secret machine learning-based algorithm aimed at helping them focus on their breath by rewarding a decrease in EEG signatures of mind-wandering (Hashemi et al., 2016). In overnight sleep recordings, users had the option to listen to various audio content and/or auditory feedback while falling asleep.

We curated four datasets by selecting recordings from InteraXon Inc.’s anonymized database of Muse users (seeTable 1) carried out in North America, South America, Europe, Asia, Africa, and Oceania. First, the*Muse Meditation Dataset*(MMD), a subset of 4191 meditation recordings from unique individuals, was obtained by sampling recordings of at least 5 minutes, with excellent signal quality, such that the age distribution was approximately uniform between 18 and 81 years of age (seeSupplementary Materials Afor the detailed procedure).

**Table 1.** Description of the datasets used in this study.

Dataset	Type	Nb of sessions	Nb of subjects	Session duration in minutes (mean ± std)	Age (mean ± std)	% female (sessions)
MMD	Meditation	4191	4191	13.88 ± 6.52	44.92 ± 14.22	39.11
MMD-long	Meditation	497	4	23.06 ± 8.72	54.51 ± 11.61	12.88
AMUSeD	Sleep	1020	1020	456.89 ± 75.05	43.78 ± 13.52	49.02
AMUSeD-long	Sleep	539	4	461.73 ± 65.62	57.64 ± 13.17	20.22

Next, the*At-home Muse Unlabelled Sleep Dataset*(AMUSeD), a subset of 1020 overnight sleep recordings from unique individuals, was obtained in a similar fashion to MMD, that is, we sampled sleep recordings of excellent signal quality, between 5 and 11 hours long, and with as uniform of an age distribution as possible (seeSupplementary Materials Bfor more details).

To study the long-term dynamics of EEG-based brain age, we additionally curated datasets containing sessions from a few users with a high number of recordings over multiple consecutive weeks and months. Following the same signal quality criteria as for MMD and AMUSeD, we sampled the*Longitudinal Muse Meditation Dataset*(MMD-long) and*Longitudinal At-home Muse Unlabelled Sleep Dataset*(AMUSeD-long), each containing recordings from four subjects (distinct subjects in both datasets) of different age groups. This yielded a total of 497 recordings for MMD-long and 539 recordings for AMUSeD-long^3^.

Of note, 138 subjects are found in both MMD and AMUSeD, there is no overlap between MMD-long and the other datasets, and three subjects are in both AMUSeD and AMUSeD-long.

### 2.3 EEG preprocessing

Minimal preprocessing steps were applied to EEG data before feeding it to the prediction models. In the case of meditation recordings, the first minute of the meditation exercise (in which signal quality might still be settling) was cropped, and as much as eight of the following minutes of data were kept, depending on the length of the recording. Sleep recordings were kept in their entirety. Next, missing values (which can occur if the wireless connection is weak and Bluetooth packets are lost) were replaced through linear interpolation using surrounding valid samples.

A zero-phase FIR band-pass filter^4^between 0.1 and 49 Hz was applied to both data types following the benchmark ofEngemann et al. (2022). We additionally applied FIR notch filters to attenuate some hardware-specific spectral peaks that appeared in some of the recordings (at 16, 21.3, 32, and 42.7 Hz) as well as power line noise frequencies (50 and 60 Hz). Though conservative, this approach to noise reduction is simple to implement and led to improved brain age prediction performance.

Non-overlapping windows were then extracted. For sleep data, we used 30-s windows, as this is the standard window length used in sleep staging (Berry et al., 2012) and, as such, allows capturing important sleep EEG microevents. For meditation data, as recordings were on average more than 57 times shorter than sleep recordings, we instead used 10 s windows to still capture longer-scale temporal dynamics but also provide enough windows for accurate covariance matrix estimation (seeSection 2.4.1). In both cases, windows for which peak-to-peak amplitude exceeded a value of 250μV were rejected. Finally, EEG time series were downsampled to 128 Hz.

### 2.4 Age prediction machine-learning pipelines

In this section, each step of the selected age prediction pipeline (Engemann et al., 2022) based on filterbank covariance matrices is described in detail.

#### 2.4.1 Filterbank covariance matrices estimation

First, a filterbank was applied to the preprocessed EEG using the coffeine package^5^, yielding narrow-band signals in nine frequency bands: low frequencies (0.1–1 Hz),δ(1–4 Hz),θ(4–8 Hz),$\alpha_{\text{low}}$(8–10 Hz),$\alpha_{\text{mid}}$(10–12 Hz),$\alpha_{\text{high}}$(12–15 Hz),$\beta_{\text{low}}$(15–26 Hz),$\beta_{\text{mid}}$(26–35 Hz), and$\beta_{\text{high}}$(35–49 Hz).

Second, spatial covariance matrices were estimated in each frequency band using non-overlapping 10 or 30 s windows. This yielded a set of nine covariance matrices$\sum^{\left(k\right)}={\mathbb{R}}^{C\times C}$per recording, wherek∈ℬ={low$\text{ frequencies},\delta ,\theta ,\alpha_{\text{low}},\alpha_{\text{mid}},\alpha_{\text{high}},\beta_{\text{low}},\beta_{\text{mid}},\beta_{\text{high}}\}$andC=4is the number of EEG channels. Covariance matrices were estimated using the Oracle Approximating Shrinkage (OAS) algorithm (Chen et al., 2010). As opposed to the empirical covariance estimator, the OAS estimator applies regularization to ensure the resulting covariance matrices are well conditioned. This yields more robust covariance matrices under noisy and limited-sample settings (Engemann & Gramfort, 2015). We additionally extracted*cross-spectral covariance matrices*to further capture interactions between channels in different frequency bands. To this end, the filtered signals in the nine frequency bands were concatenated before computing their covariance matrix, yielding a single cross-spectral covariance matrix$\sum_{cs}={\mathbb{R}}^{C\left|ℬ\right|\times C\left|ℬ\right|}$per recording.

#### 2.4.2 Vectorization

Next, we applied a vectorization operation to flatten (sets of) covariance matrices into feature vectors$\nu \in {\mathbb{R}}^{F}$to be fed to the machine-learning model${\text{f}}_{\Theta }$. We considered two vectorization approaches:*log-diagonal extraction*and*Riemaniann tangent space projection*(Sabbagh et al., 2019).

##### 2.4.2.1 Log-diagonal extraction

In the first case, we extracted the log-diagonal of each covariance matrix before concatenating the diagonals of all nine matrices:

$$\nu \in {\mathbb{R}}^{C\left|ℬ\right|}=\underset{k\in ℬ}{\left|\right|}\text{log}\left(\text{diag}\left(\sum^{\left(k\right)}\right)\right),$$ (1)

where∥is the concatenation operator anddiagextracts the diagonal of a square matrix. This is equivalent to using the set of channel-wise log-powers in the nine frequency bands ofℬ.

##### 2.4.2.2 Riemannian tangent space projection

In the second case, covariance matrices were instead projected into their Riemannian tangent space, exploiting the Wasserstein distance to estimate the mean covariance matrices$\bar{\Sigma^{\left(k\right)}}$used as reference points (Bhatia et al., 2019;Sabbagh et al., 2019)^6^. Denoting the matrix square root of a covariance matrix as$\sum =\text{Y}{\text{Y}}^{⊺}$, the vectorized features are computed as follows:

$$\nu \in {\mathbb{R}}^{\left(C\left(C+1\right)/2\right)\left|ℬ\right|}=\underset{k\in ℬ}{\left|\right|}\text{vect}\left({\text{Y}}^{\left(k\right)}{\text{Q}}^{\text{*}}-\bar{{\text{Y}}^{\left(k\right)}}\right),$$ (2)

where thevectoperator flattens a matrix into a vector of all its elements,${\text{Y}}^{\left(k\right)}$and$\bar{{\text{Y}}^{\left(k\right)}}$are the matrix square root of the input and mean covariance matrices, respectively, and${\text{Q}}^{\text{*}}\text{​}=\text{V}{\text{U}}^{⊺}$given the singular value decomposition of${\text{Y}}^{\left(k\right)}{\bar{{\text{Y}}^{\left(k\right)}}}^{⊺}\text{​}=\text{UD}{\text{V}}^{⊺}$.

In total, this led to three variations over the basic filterbank covariance matrix pipeline, as described inTable 2. The*spectral*model is called as such because it only contains the log-power of each channel in each frequency band, but no cross-channel information. The*spectro-spatial*model, thanks to the different vectorization approach, contains information about the covariability of pairs of channels in each frequency band. Finally, the*cross-spectro-spatial*model further allows capturing interactions between channels in different frequency bands.

**Table 2.** Description of the different age prediction model variants along with the resulting number of features forC= 4 EEG channels and|ℬ|= 9 frequency bands.

Model name	Covariance matrix type	Vectorization	Nb of features (meditation)	Nb of features (sleep)
*Spectral*	Spatial covariance in each band	Log-diagonal	36	180
*Spectro-spatial*	Spatial covariance in each band	Riemaniann tangent space	90	450
*Cross-spectro-spatial*	Cross-spectral covariance	Riemaniann tangent space	666	3330

##### 2.4.2.3 Sleep-specific vectorization

To train models on sleep recordings, the pipeline was slightly modified such that one set of covariance matrices was extracted per sleep stage (W, N1, N2, N3, R). By representing each sleep stage by its own set of features, we ensured that stage-specific spectral and spatial characteristics were made available to the machine-learning models. We used an automatic sleep staging model (Abou Jaoude et al., 2020) trained on a labelled subset of Muse S overnight recordings to obtain sleep stage predictions for each 30 s window of AMUSeD and AMUSeD-long recordings (seeSupplementary Materials Cfor a detailed description of the sleep staging pipeline). We used this specific architecture because it provided good performance on data similar to the one contained in AMUSeD in preliminary testing; however, other generic sleep stage classifiers could have been used instead, for example, a feature-based model such as inVallat and Walker (2021)or a different deep learning architecture such as inPhan et al. (2022). Filterbank covariance matrices were then extracted and vectorized per sleep stage following the same procedure as described above, before concatenating all resulting feature vectors into a single vector.

#### 2.4.3 Regression model

The vectorized features were finally z-score normalized using the mean and standard deviation of the training set, before being fed to a Ridge regression model, that is, a linear regression model with an$\ell_{2}$penalty (Hoerl & Kennard, 1970). Ridge regression is a commonly used method for EEG-based regression tasks (Congedo et al., 2017;Engemann et al., 2022;Sabbagh et al., 2020).

#### 2.4.4 Evaluation and metrics

Models were trained on MMD or AMUSeD using Monte Carlo (shuffle split) cross-validation with 100 splits and 10% testing data. The training folds were further split to perform a hyperparameter search using generalized leave-one-out cross-validation for selecting the regularization strength. The regularization parameter was chosen among 100 values spaced log-uniformly between$10^{-5}$and$10^{10}$. The examples from each subject were always used in only one of the training, validation or testing sets.

Predictions on the test folds were then used to evaluate the performance of the different models, as measured with the coefficient of determination$R^{2}$(Hughes & Grawoig, 1971), that is, the percentage of chronological age variance explained by brain age:

$$R^{2}\left(\text{y},\hat{\text{y}}\right)=1-\frac{\sum_{i=1}^{N}{\left(y^{\left(i\right)}-{\hat{y}}^{\left(i\right)}\right)}^{2}}{\sum_{i=1}^{N}{\left(y^{\left(i\right)}-\bar{y}\right)}^{2}},$$ (3)

where$\bar{y}=\frac{1}{N}\sum_{i=1}^{N}y^{\left(i\right)}$. We also used the mean absolute error (MAE) as an additional measure of age prediction performance:

$$\text{MAE}\left(\text{y},\hat{\text{y}}\right)=\frac{1}{N}\sum_{i=1}^{N}\left|y^{\left(i\right)}-{\hat{y}}^{\left(i\right)}\right|.$$ (4)

##### 2.4.4.1 Longitudinal evaluation

Finally, models were retrained using the entire MMD or AMUSeD as training set (with the same validation set splitting strategies) and evaluated on the longitudinal recordings of MMD-long or AMUSeD-long.

### 2.5 Computational considerations

A combination of the MNE-Python (Gramfort et al., 2014), pyRiemann (Barachant et al., 2013), coffeine (Sabbagh et al., 2020), mne-features (Schiratti et al., 2018), scikit-learn (Pedregosa et al., 2011), mne-bids (Appelhoff et al., 2019), and mne-bids-pipeline (Jas et al., 2018) packages were used to carry out our experiments.

## 3 Results

### 3.1 Predicting age from at-home mobile EEG recordings

Can low-cost, at-home mobile EEG be used to predict someone’s age, even if EEG is recorded with very few channels in environments that are not experimentally controlled? To explore this question, we trained machine-learning models to predict age from such at-home EEG data on thousands of meditation or overnight sleep recordings. The performance obtained by these models is shown inFigure 1. Scatter plots showing the relationship between chronological age and predicted brain age are shown in Supplementary materials D (Fig. S2).

**Fig. 1.**
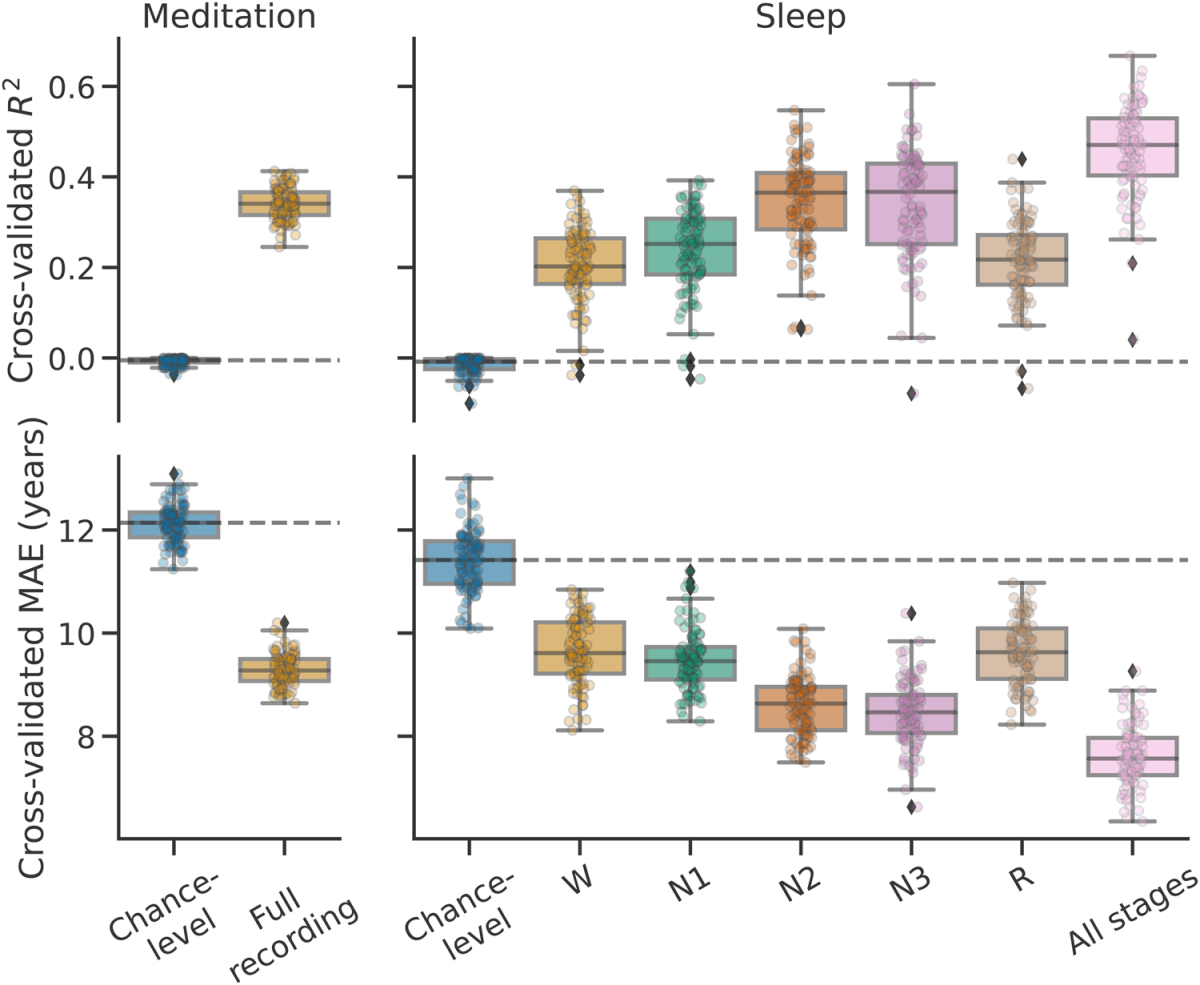
Cross-validated brain age prediction performance (first row:$R^{2}$, second row: MAE) of models trained on at-home meditation or overnight sleep mobile EEG recordings (Monte Carlo, 100 iterations, 10% testing data). Points represent the scores of individual models trained on different cross-validation splits, while the boxplots display the overall score distributions.**Left column**: Models trained and evaluated on 4191 4- to 8-minute recordings of an eyes closed meditation exercise performed better than chance.**Right column**: Individual models trained on a dataset of 1020 overnight sleep recordings, using a single sleep stage (W, N1, N2, N3, or R) or all stages at once (“All stages”) also performed better than chance.

Models trained on meditation data (Fig. 1, left) achieved a median$R^{2}$of 0.34 (MAE = 9.27). This is markedly above chance-level performance as estimated by evaluating a dummy predictor which always predicts the median age in the training set. Such a dummy regressor yields a median of -0.01$R^{2}$(12.14 MAE) on meditation data. Similarly, on sleep data (Fig. 1, right), all models achieved above-chance performance, with models trained on all sleep stages obtaining the highest performance (median$R^{2}$of 0.47 vs. -0.01 chance-level; median MAE = 7.57 vs. 11.42 chance-level). The performance of models trained on either W or R stages alone was the lowest (0.20 and 0.22$R^{2}$; 9.61 and 9.63 MAE), while N2- and N3-based models both achieved higher performance (both 0.37$R^{2}$; 8.63 and 8.46 MAE). Performance was highest when combining information across all sleep stages, suggesting that additive information was conveyed by different sleep stages. Does this finding imply that sleep is even better suited for age prediction than meditation? While the results do show higher$R^{2}$for sleep-based models, the values cannot be directly compared as the age distribution, recording duration, and sample sizes of the meditation and sleep datasets are not the same (as shown by different chance-level mean absolute error distributions, seeFig. 1, second row).

In sum, these findings show that machine-learning models initially developed for the laboratory setting can accurately predict an individual’s age based on their EEG collected with an at-home mobile device from either sleep or meditation contexts.

### 3.2 Inspection of trained brain age models

What drives the predictions of these models, that is, what kind of information is critical for predicting age? Knowing this would not only validate the functioning of our models, but also provide interesting information to identify markers of aging in the human brain. To answer this question, we trained additional brain age models on the meditation and sleep datasets, but this time with inputs that capture different types of information. Results are presented inFigure 2.

**Fig. 2.**
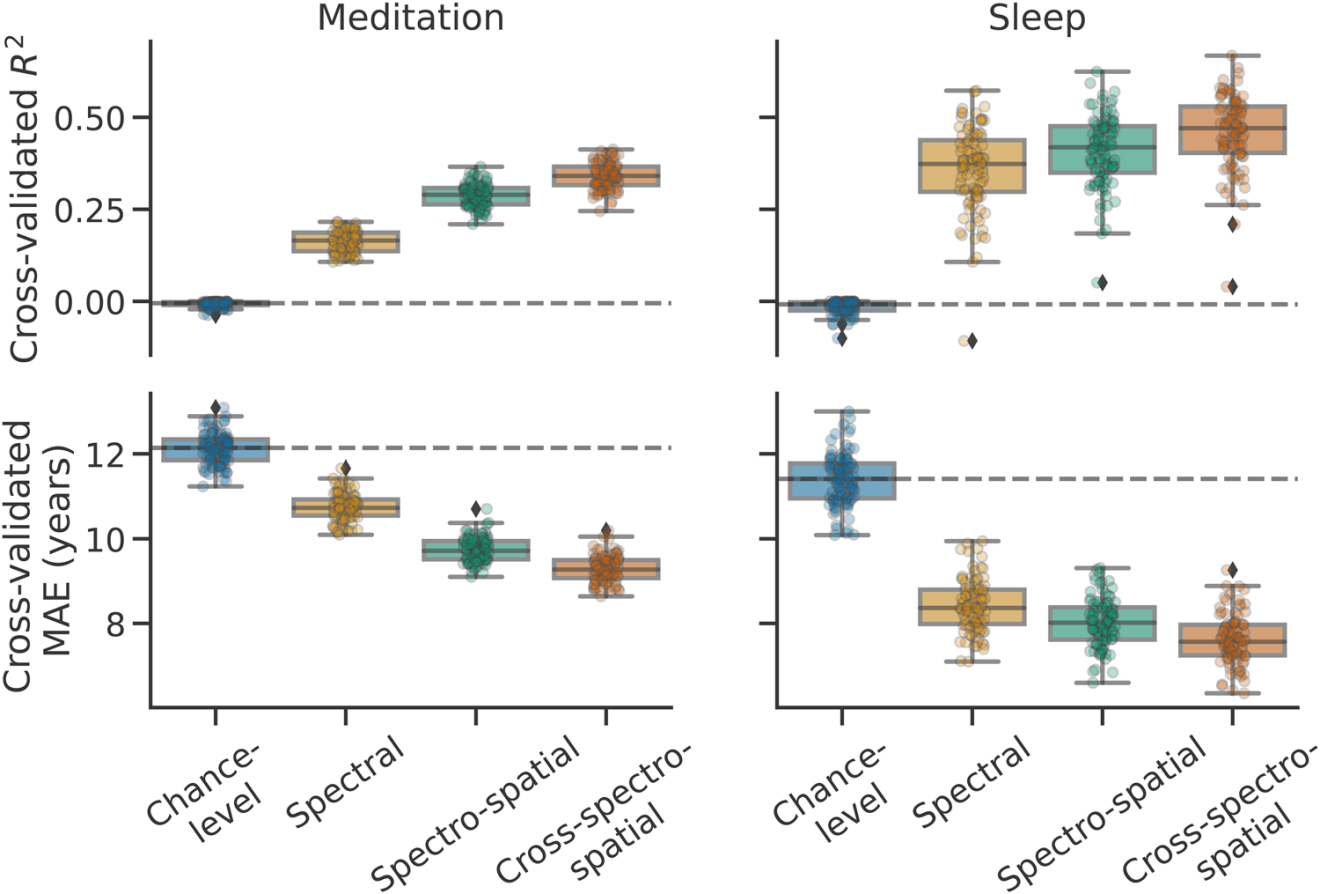
Cross-validated brain age prediction performance (first row:$R^{2}$, second row: MAE) of models trained on different EEG input representations (Monte Carlo, 100 iterations, 10% testing data) for (**left column**) meditation and (**right column**) sleep recordings. For sleep results, models based on all sleep stages (“All stages” inFig. 1) were used. For both meditation and sleep data, the inclusion of spectral, spatial, and cross-spectral information yielded the best performance despite the limited number of only four EEG channels.

For both meditation- and sleep-based models, providing spectral information only (i.e., frequency band log-powers for each EEG channel) already yielded performance visibly above chance. Adding further spatial information, that is, by allowing the model to learn from the correlation between pairs of channels, yielded a clear improvement for both data types, with 100/100 and 85/100 of the trained “spectro-spatial” models reaching better performance across cross-validation iterations than their equivalent “spectral”-only models for meditation and sleep data, respectively. This suggests that spatial information might be slightly more useful for decoding age from meditation rather than from sleep data. Finally, additionally allowing the models to learn from cross-frequency correlations helped improve performance for both meditation and sleep models, that is, cross-spectro-spatial models were better than spectro-spatial models on 99/100 and 96/100 cross-validation iterations, respectively.

**Fig. 3.**
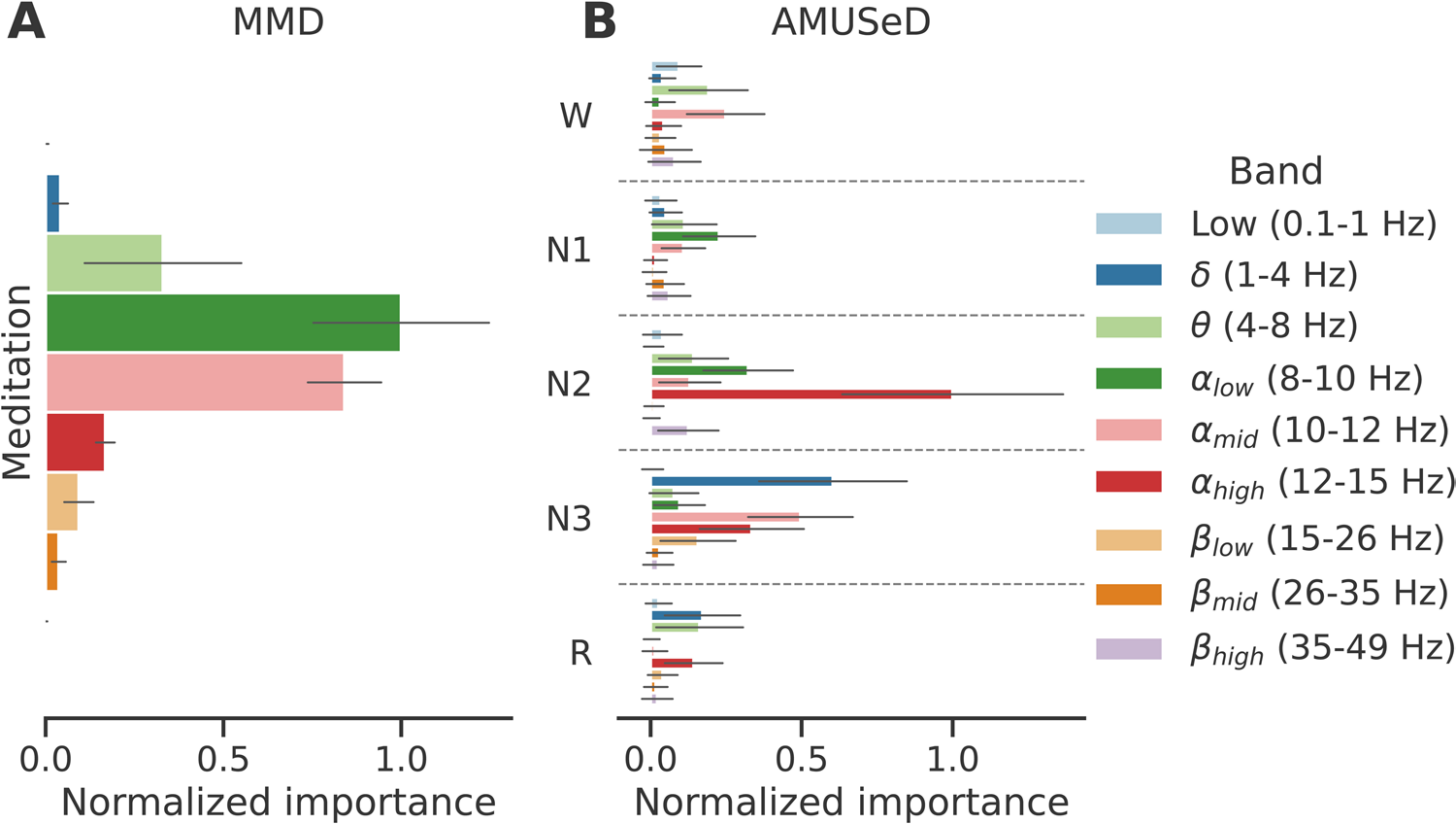
Permutation analysis using the spectral models trained on (A) meditation and (B) sleep data. The x-axis indicates the average performance variation ($\Delta R^{2}$, normalized within dataset by the highest band-wise average importance value for comparability) obtained when the values in a specific frequency band or a combination of a frequency band and sleep stage are randomly permuted over 100 repetitions at testing time. Error bars indicate standard deviation across permutations. On meditation data, models were most sensitive to$\alpha_{\text{low}}$and$\alpha_{\text{mid}}$bands, while on sleep data models were most sensitive to$\alpha_{\text{high}}$in N2 andδand$\alpha_{\text{mid}}$in N3.

Given that strictly spectral-based models already yielded above-chance age prediction performance, we can further inspect them to see which frequency bands provided the most useful information. As shown by a permutation importance analysis^7^(Breiman, 2001), different bands were useful for the meditation- and sleep-based models (Fig. 3). On meditation data, low and midαsub-bands (8–10 and 10–12 Hz) were the most important, that is, making this information unusable by the models resulted in the largest performance decrease. On sleep data, given that the spectral information was extracted per sleep stage, it is possible to consider the importance of the different bands in each sleep stage (Fig. 3B). Strikingly, the most useful feature was$\alpha_{high}$(12–15 Hz) in N2, whose importance was about twice as high as the second most useful feature,δpower (1–4 Hz) in N3. Moreover, mid and highα(10–12 and 12–15 Hz) in N3 also stood out as critical features. The frequency ranges and sleep stages identified above relate to well-known characteristics of awake and sleep EEG (e.g., alpha peak, sleep spindles, and slow waves), suggesting that the models picked up biological information.

Overall, these results suggest that spectral, spatial, and cross-spectral dimensions all provide complementary information that can help predict an individual’s age from their mobile EEG. By evaluating the importance of the different frequency bands, we further demonstrated that our models relied on specific EEG patterns to predict age, providing initial validation of our approach.

### 3.3 Variability of brain age over multiple weeks and months

To assess the practicality of the brain age metric obtained from home-based EEG with few electrodes, it is helpful to consider its variability over medium- to long-term periods. Indeed, stable values over weeks and months would indicate actual “trait”-level information, for example, related to aging and, potentially, pathological aging, is being captured. On the other hand, significant variability at smaller scales would suggest the metric is also influenced by momentary factors and therefore captures “brain states” as well. To answer these questions, we computed the recording-by-recording brain age of a few subjects for whom a large number of recordings was available and inspected their predicted brain age Δ values. The results of this analysis are presented inFigure 4(seeFig. S4for additional results).

**Fig. 4.**
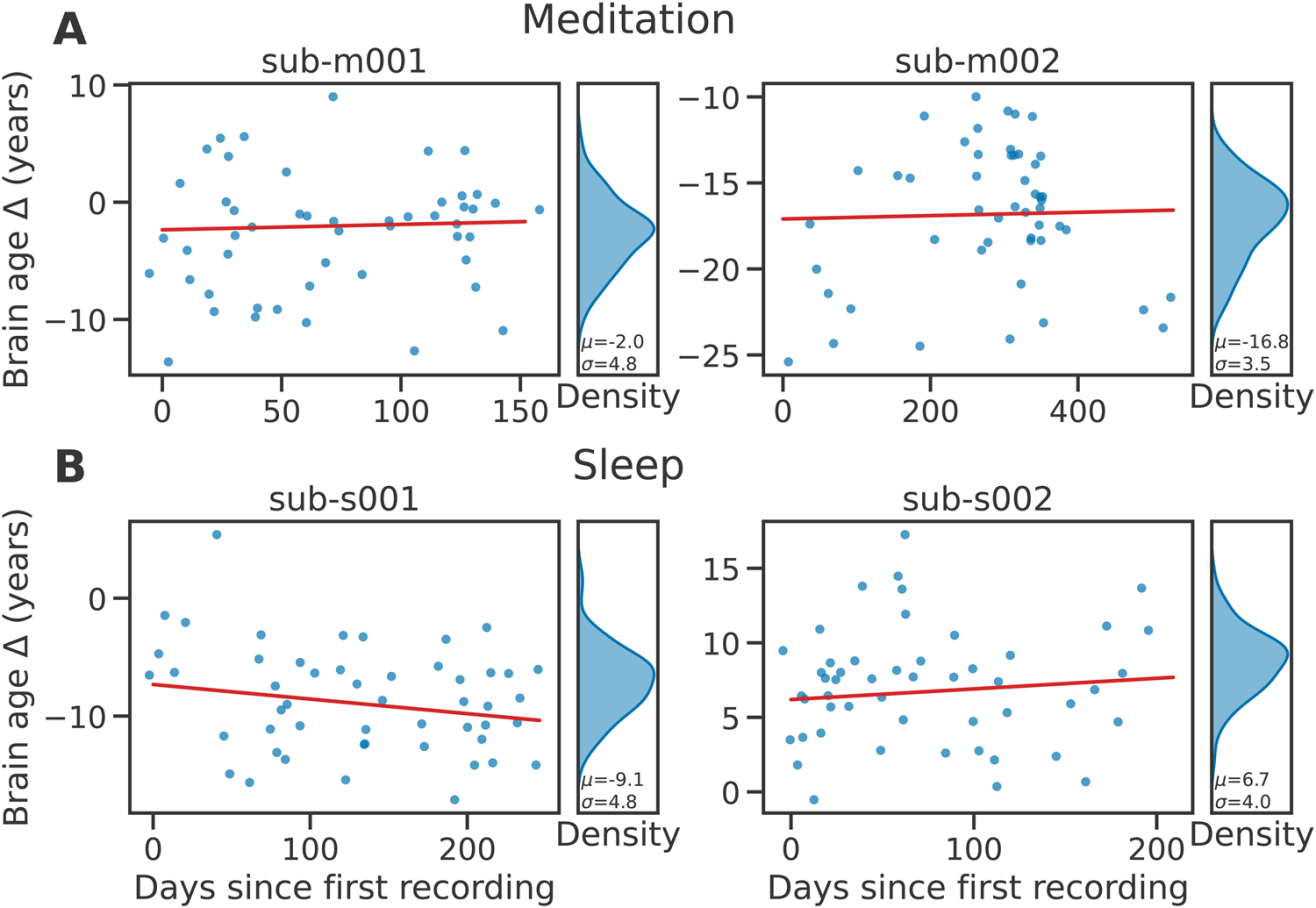
Longitudinal brain age Δ predictions for four subjects with (A) multiple consecutive meditation or (B) sleep recordings. Four subjects with more than 50 recordings of good signal quality were selected. Their brain age was predicted using a model trained on MMD or AMUSeD. Blue dots represent the brain age Δ predicted for single recordings. To ensure the anonymity of the subjects, we only presented 50 randomly sampled recordings for each subject and added random jitterδ∼N(0,20 days)to the recording dates. Nevertheless, all available sessions were used to fit a linear model (red line) showing the trend for each subject. Density plots summarize the distribution of predicted age (blue marginal plots). Despite significant variability visible in all subjects, the slopes obtained from linear models across sessions (red lines) were close to zero, pointing at a stable average brain age across time. This suggests the proposed brain age metric captures both “trait”- and “state”-like information.

While there is substantial longitudinal variability across recordings from a same subject, the average predicted values remain fairly stable across longer-term periods, for both meditation- and sleep-based models. Despite across-recording standard deviations of 3.5 to 6.0 for meditation, and 3.3 to 5.5 for sleep, the average predictions over longer periods (e.g., months) do not vary substantially as can be seen from the relatively flat slopes obtained from linear models (min=−0.017,max=0.015). This is seen both in subjects whose chronological and brain age match closely (e.g., sub-m001) and in subjects for whom there is a larger difference between the two measures (e.g., sub-m002). Overall, the within-subject standard deviation in brain age Δ remained lower in longitudinal datasets than the cross-subject standard deviation in large cross-sectional datasets (mean of 4.8 years vs. 11.6 years for MMD-long and MMD, and mean of 4.4 vs. 9.9 for AMUSeD-long and AMUSeD).

Taken together, these results suggest that our brain age metric derived from mobile EEG captures “trait”-like information, but also shorter-term “state”-like information which may reflect a subject’s state at the time of the recording.

## 4 Discussion

In this paper, we showed that brain age measurement, thus far restricted to clinical and laboratory settings, can be extended to at-home settings using low-cost mobile EEG. We presented experiments on over 5200 recordings from unique individuals, combining real-world data collected during meditation exercises and overnight sleep. Results highlighted the usefulness of cross-spatio-spectral information to predict age, and revealed how certain EEG characteristics, such as$\alpha_{low}$power for meditation, N2$\alpha_{high}$power in the spindle range, and N3δpower, are important for accurately predicting age. Inspired by how mobile EEG technology could facilitate longitudinal monitoring of brain biomarkers, we investigated the stability of brain age over repeated sessions spanning a few months to over a year for eight individuals. This exploration revealed short-term recording-to-recording variability but also stability across longer-term periods that have not been reported in the previous literature focusing on very few or single snapshots of individual EEG (Banville et al., 2021;Engemann et al., 2022;Sabbagh et al., 2020;Sun & Saenko, 2016). Our results provide a foundation for the development of brain age assessment tools that can be applied out-of-the-lab with real-world EEG.

### 4.1 Biomarker measurement in real-world conditions with mobile EEG

The results presented in this work were obtained on recordings carried out at home by users themselves, without expert supervision or a complicated experimental setup, relying instead on off-the-shelf, low-cost mobile EEG hardware. Because it is both an easier and cheaper way to record data, this approach could enable collecting larger and more diverse samples. Concretely, we trained models on datasets containing more than 5200 of at-home EEG recordings (seeTable 1). This is an order of magnitude above much of the published work on EEG-based brain age and in line with the previous studies with the highest sample sizes (Brink-Kjaer et al., 2022;Engemann et al., 2022;Sun et al., 2019). Critically, these studies focused on clinical or research datasets which are much more expensive and time-consuming to collect. Despite working on noisier real-world EEG data, brain age prediction performance was in a similar range as these published results, for example, 0.47 versus 0.33-0.61$R^{2}$inEngemann et al. (2022)on their largest reported dataset, and 7.6 (Fig. 1, second row, “All stages”) versus 7.8 years mean absolute error inSun et al. (2019). Likely, this similarity in performance reflects the fact that sleep EEG is a rich source of physiological information and that a few channels are sufficient to capture some of its age-related features well. Moreover, our results support the use of a prediction pipeline based on filterbank covariance matrices and Riemannian geometry, as previously proposed for brain age modeling (Engemann et al., 2022;Sabbagh et al., 2020,^2023^).

The ease-of-use of mobile EEG devices also make it easier to collect longitudinal data over extended periods of time. This has allowed us to evaluate brain age longitudinally on an unprecedented number of repeat sessions, that is, 1036 recordings from four subjects spanning periods of up to 529 days, while previous work was limited to a few days, for example, four recordings collected over consecutive days or a few days apart (Hogan et al., 2021). This highlights the potential of mobile EEG to bring biomarker monitoring out-of-the-lab and into the real world.

### 4.2 What does EEG-based brain age capture?

Brain age models trained on structural MRI data rely on anatomical features such as cortical thickness and brain volume to provide accurate predictions of one’s chronological age (Cole et al., 2019). As EEG captures brain activity, rather than anatomy, what specific information does it contain that enables age prediction? Given the important differences between awake and sleep EEG, the answer differs for the types of data presented in this study.

Sleep EEG provided higher age prediction performance than awake EEG (seeFig. 1). This was not entirely surprising, as there is a well-known connection between sleep microstructure and age (Muehlroth & Werkle-Bergner, 2020;Purcell et al., 2017), meaning the predictive models have access to rich age-related information (through the cross-spectro-spatial representation described inSection 2.4). This is in line with the results ofFigure 3which revealed that the most useful features for predicting age are in fact descriptors of key sleep microstructure components. For instance,$\alpha_{high}$(12–15 Hz) power in N2, the most important feature according to the analysis, fits with theσband (11–15 Hz) sleep spindles typically fall in (Purcell et al., 2017). The second most useful feature,δ(1–4 Hz) power in N3, captures the well-documented age-related decrease in slow wave (<4 Hz) power (Muehlroth & Werkle-Bergner, 2020). Nonetheless, combining information from all sleep stages yielded the best age prediction performance (seeFig. 1), suggesting there is additional useful information that might not be strictly related to well-known microevents. Similarly, including data from the entire night, rather than from the beginning or the end, leads to better performing models, as seen inFig. S3of Supplementary materials D. These results confirm overnight sleep EEG is a rich source of information for modeling brain age. Of note, it might be possible to reduce the gap between meditation- and sleep-based age prediction performance by pooling information from more than one awake state, that is, by combining multiple types of meditation exercises or recording data under varied cognitive paradigms.

Despite lower performance, models based on awake meditation EEG also yielded reasonable brain age predictions. Previous work on very similar cross-sectional data but from a descriptive, rather than predictive, point of view has shown that the characteristics of theαband vary significantly throughout adulthood (Hashemi et al., 2016). For instance, theαpeak frequency decreases with age (a widely reported finding, see, e.g.,Merkin et al. (2023);Chiang et al. (2011);Knyazeva et al. (2018);Klimesch (1999)). Additionally, a small but significant increase inαandβpower with age has also been reported in conjunction with a decrease inδandθpower (Hashemi et al., 2016). Indeed, our filterbank models heavily relied on the information contained in the low- and mid-αbands, that is, between 8 and 12 Hz (seeFig. 3). As shown inFig. S5of Supplementary materials E, visualization of the spectrum around theαband similarly confirmed that the peakαfrequency decreased across age groups.

Overall, brain age models based on mobile EEG can capture known age-related electrophysiological phenomena. This provides early support for extending brain age measures beyond anatomical and lab-based assessments. Interestingly, the link between anatomical features such as cortical thickness and EEG-derived markers has been studied in past studies, though not in the specific context of brain age prediction. For instance, previous studies have linked cortical thinning with decreasedαpower (Bruder et al., 2012), high$\alpha_{high}$/$\alpha_{low}$power ratio (Moretti et al., 2013), and changes inδband power during sleep (Latreille et al., 2019). On the other hand, previous work on brain-age prediction from MEG (Engemann et al., 2020;Sabbagh et al., 2020;Xifra-Porxas et al., 2021) found that MEG and MRI provided, both, overlapping (via volume conduction) and complementary information, potentially, related to patterns of large-scale cortical dynamics (Engemann et al., 2020;Hipp et al., 2012). Investigating the precise connection between anatomical and functional measures might further help establish the EEG correlates of brain age predictions in future work.

### 4.3 What causes brain age variability?

Longitudinal monitoring on eight subjects showed that while the average brain age Δ predictions were mostly stable across longer periods of time (e.g., months), there was significant variability from one day to the next. Of note, another study which looked at EEG-based brain age across days, though on a shorter temporal scale, found a night-to-night standard deviation in brain age Δ of 7.5 years (Hogan et al., 2021). This standard deviation could be further decreased to 3 years when averaging four nights. In comparison, our experiments on sleep data (seeFig. 4and S4) showed a standard deviation of 3.3 to 5.5 years for four subjects with at least 50 recordings each. Additional experiments on more subjects will be required to validate these results, however, this already suggests that similar variability is observed even with longer sleep EEG recordings. While a supplementary analysis (Supplementary Materials F) suggests that time of day, at least cross-sectionally, is negatively correlated with brain age Δ (Pearson’sρ=−0.079,$p<10^{-5}$), most of the variability remained unexplained. What does this residual variability reflect?

On sleep data, intrinsic factors, for example, fluctuations in sleep quality, likely account for some of this variability. For instance, increased slow wave activity (Plante et al., 2016) and decreased sleep spindle density but increased amplitude (Knoblauch et al., 2003) are established effects of sleep deprivation. As our models rely onδpower in N3 to predict age andσpower in N2, the fluctuations might in fact partially reflect sleep quality or even past sleep dept. Similarly, for awake meditation data, different factors are known to influence theαpeak, whose characteristics were most important for predicting age in our sample. For instance, it has been suggested that a higher peakαfrequency is associated with a higher level of “cognitive preparedness”, that is, reflecting how ready an individual is to perform a task (Angelakis et al., 2004;Christie et al., 2017). Research on “mental fatigue” has also revealed that the spectral properties of EEG change as fatigue increases with, for instance, power inθ,α, andβbands increasing (Arnau et al., 2017). Multiple studies on meditation and EEG also reported decreasedαpeak frequency and increasedαpower during meditation exercises (Cahn & Polich, 2006;Lomas et al., 2015). The observed variability might therefore reflect the quality of the meditation, the specific type of meditation exercise carried out during the recording, or overall well-being related to recovery from activities during the day before the recording.

The observed patterns might appear inconsistent with the expected stability of an age-related measure, that is, to be evolving slowly in time. However, it is important to recall that because existing brain age studies are typically limited to single or very few recordings per subject, similar fluctuations between recordings may simply be unknown. On the other hand, one could hypothesize that high-density EEG and a focus on EEG features beyond spectral power, for example, measures of connectivity, may improve re-test reliability (Rolle et al., 2022). Ultimately, longitudinal analysis over even longer time scales (e.g., multiple years) and models that include additional factors such as heritability (Vandenbosch et al., 2019) will be necessary to properly characterize the causes of brain age variability. Unless head-to-head comparisons between mobile devices and laboratory-grade EEG are performed, this remains speculative. But in case future studies will uncover strong intrinsic variability between recordings across device types, mobile EEG assessments would have a clear advantage. Performing multiple repeat measures to estimate trait-like signatures will be far more tractable with home-based EEG.

### 4.4 Limitations

The datasets used in our experiments were collected in uncontrolled, at-home conditions, that is, no expert monitoring was available to ensure compliance with the recording instructions. This contrasts with existing work on brain age prediction, where datasets are usually collected in clinical or research settings. Similarly, only minimal metadata were available in the datasets. As a result, it was not possible to know whether some individuals contained in our datasets suffered from medical conditions. Commonly, in brain age studies, data from healthy individuals are used for training and data from pathological individuals are kept for testing. Instead, as our models, during training, were possibly exposed to some data from people suffering from medical conditions, the sensitivity of a resulting brain-age metric to pathology-related EEG activity might be lower than in previous studies realized in which models were trained on healthy participants and applied to brain data from clinical settings. Likewise, we did not have access to clinical cognitive assessments that would have allowed exploration and validation of brain age semantics, for example, relating positive brain age Δ to cognitive slowing (Denissen et al., 2022;Engemann et al., 2020). Finally, the datasets were not collected under controlled conditions typical of laboratory- or clinical-based studies, leaving open the possibility that individuals did not enter their actual age when creating their user profile. However, thanks to the type of sample size enabled by mobile EEG devices, much larger than traditional studies, our models will be less sensitive to the occasional incorrect label. For instance, a study using similar datasets produced results that agreed with earlier lab-based studies (Hashemi et al., 2016), supporting the validity of our dataset for brain age analysis. Similarly, because the recordings we analyzed have limited spatial coverage, age prediction studies on laboratory or clinical data remain necessary to ensure a frontal and parietal montage such as the one used here offers a reasonable trade-off between ease-of-use and prediction accuracy.

## 5 Conclusion

Despite the challenges that come with out-of-the-lab collection of EEG, individual physiological characteristics can be successfully modeled during sleep and meditation using state-of-the-art machine learning. Brain age prediction, in particular, is a promising framework to leverage large-scale real-world EEG data. Combined with these tools and thanks to its cost-effectiveness and ease of use, at-home low-cost mobile EEG offers a promising opportunity to democratize, accelerate, and scale up biomarker development.

## Supplementary Material

Supplementary Material

## Data Availability

EEG recordings were collected on users of the Muse S headband according to InteraXon’s privacy policy (https://choosemuse.com/legal/privacy/). According to this policy, recordings cannot be shared unless a formal data sharing agreement has been put in place with InteraXon Inc. The analysis code can be reproduced using the supporting code for the systematic benchmark of brain age prediction pipelines ofEngemann et al. (2022)(https://github.com/meeg-ml-benchmarks/brain-age-benchmark-paper).

## Footnotes

1
https://choosemuse.com/legal/privacy/

2
https://choosemuse.com/muse-app/

3 We do not disclose these subjects’ ages or gender in order to minimize identifiable information.

4 Zero-phase filtering was used given the offline nature of the age prediction task and as it is the default setting in the mne-bids-pipeline and MNE-python packages, though a causal filter could have been used instead.

5
https://github.com/coffeine-labs/coffeine

6 We used the Wasserstein distance instead of the geometric distance as it performed better in preliminary experiments. Interestingly, this might be due to the Wasserstein distance being more robust to poorly conditioned covariance matrices that arise when working with noisy data such as real-world EEG (Banville et al., 2022) as the square root of zero (practically encountered in eigenvalues with small numbers) is defined whereas the log is not. This model is consistent with a square-root non-linearity between the EEG source powers and the target of interest, that is, subject age. This may or may not reflect the true underlying generator but can offer a reasonable approximation given the nature of real-world EEG data collected from wearable sensors. Importantly, the Wasserstein distance showed similar performance for age-prediction compared to the geometric distance inSabbagh et al. (2019).

7 The permutation analysis measures how the model’s performance, measured as$R^{2}$, changes when one of its features has been randomly shuffled across the examples of the test set. Here, we normalized the$\Delta R^{2}$values by the highest band-wise average value for each dataset so that feature importance was straightforward to compare between the two datasets.
