# Supplementary material for: Do try this at home: Age prediction from sleep and meditation with large-scale low-cost mobile EEG
Source: Imaging Neurosci (Camb). 2024 Jun 21;2:imag-2-00189. doi: 10.1162/imag_a_00189 (PMC12272215; doi:10.1162/imag_a_00189)
Supplement: Supplementary Material [file imag_a_00189-supp.pdf]

Supplementary materials for "Do try this at home: Age prediction from sleep and meditation with large-scale low-cost mobile EEG"

A Sampling of the Muse Meditation Dataset (MMD)

Meditation recordings of five minutes and more were selected from Muse S meditation recordings collected between October 2019 and October 2021. Recordings were then filtered to only keep users whose age was between 18 and 81 years at the time of recording. A single recording was sampled per user, such that the age distribution across all sampled recordings was approximately uniform and the dataset was balanced for male and female users. From this set, only recordings with excellent signal quality, based on basic signal statistics defined as follows, were retained. First, recordings for which more than 5% of samples were missing for any of the EEG channels (caused by Bluetooth packet loss during transmission from the headband to the mobile device) were rejected. The variance of the signals bandpass-filtered between 2 and 26 Hz was also computed for non-overlapping 1-s windows. Recordings for which, for any of the four channels, 25% or more of the windows had a variance above a threshold of 100 μV^2 were rejected. This finally yielded a subset of 4191 recordings (mean duration: 13.88 ± 6.52 minutes) with excellent signal quality from 4191 unique individuals. Mean age across recordings was 44.92 ± 14.22 years old (min: 18, max: 81) and 39.11% of recordings were of female users (see Fig. S1 for more details on the age distribution).

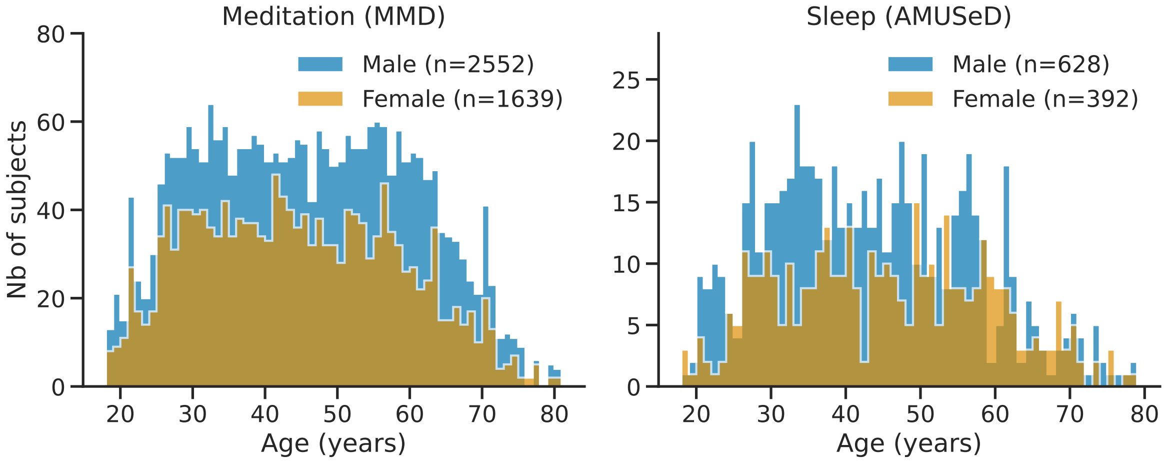

Figure S1: Age distribution for meditation recordings of MMD (left) and sleep recordings of AMUSeD (right).

B Sampling of the At-home Muse Unlabelled Sleep Dataset (AMUSeD)

We sampled sleep recordings, following a similar procedure to the one described above for MMD. Recordings that lasted between 5 and 11 hours, from users between 18 and 81 years of age at the time of recording, and which were started between 5 PM and 5 AM, local time, were selected. A maximum of two recordings per user were selected, which were then screened for signal quality. Signal quality screening was performed on non-overlapping 30 s windows (*i.e.,* the standard window length in sleep recording analysis). Recordings for which more than 25% of the windows had a variance above 1,000 μV^2 were rejected. Finally, the recording with the highest number of good windows was kept for each user. This yielded a total of 1020 overnight sleep recordings from 1020 unique users, with a mean duration of 456.89 ± 75.05 minutes, a mean age across recordings of 43.78 ± 13.52 years (min: 18, max: 79) and 20.22% of recordings of female users (see Fig. S1 for more details on the age distribution).

C Automatic sleep staging

In order to train brain age models on sleep stage-specific features, we obtained sleep stage predictions using a classifier trained on labeled Muse S data. We used the convolutional and recurrent neural network *MuseNet* model described in Abou Jaoude et al. (2023) trained on Muse S sleep recordings annotated according to the AASM guidelines (Berry et al., 2012) by a sleep technician. In contrast to the architecture originally described in Abou Jaoude et al. (2020), the updated model of Abou Jaoude et al. (2023) 1) used unidirectional, rather than bidirectional, LSTM layers, to allow its use in a real-time processing context and 2) had different input layer and maxpooling kernel sizes, in order to accommodate signals sampled at 128 Hz (vs. 200 Hz) and used a Dynamic Spatial Filtering module (Banville et al., 2022) before the first layer. The model was trained to predict which sleep stage (W, N1, N2, N3 or R) a 30 s EEG window corresponded to. Moreover, the data was preprocessed in a similar manner to what is described in Section 2.3: 1) linear interpolation of missing values, 2) downsampling to 128 Hz, 3) bandpass filtering between 1 and 40 Hz, and 4) channel-wise zero-meaning of each window.

The sleep recordings used for training the sleep staging model and those of AMUSeD (used for training the sleep-based age predictors) came from the same large pool of Muse S recordings but were curated independently as part of separate research projects. In total, 7 of the 1020 recordings of AMUSeD were also used in the training set of the sleep staging dataset (1 in validation set and 1 in test set). While this makes it possible that sleep stage predictions for these 7 sessions were biased (towards the ground truth sleep stages), we expect the effect to be negligible given the large sample size of AMUSeD and the use of Monte Carlo CV to train and evaluate our models.

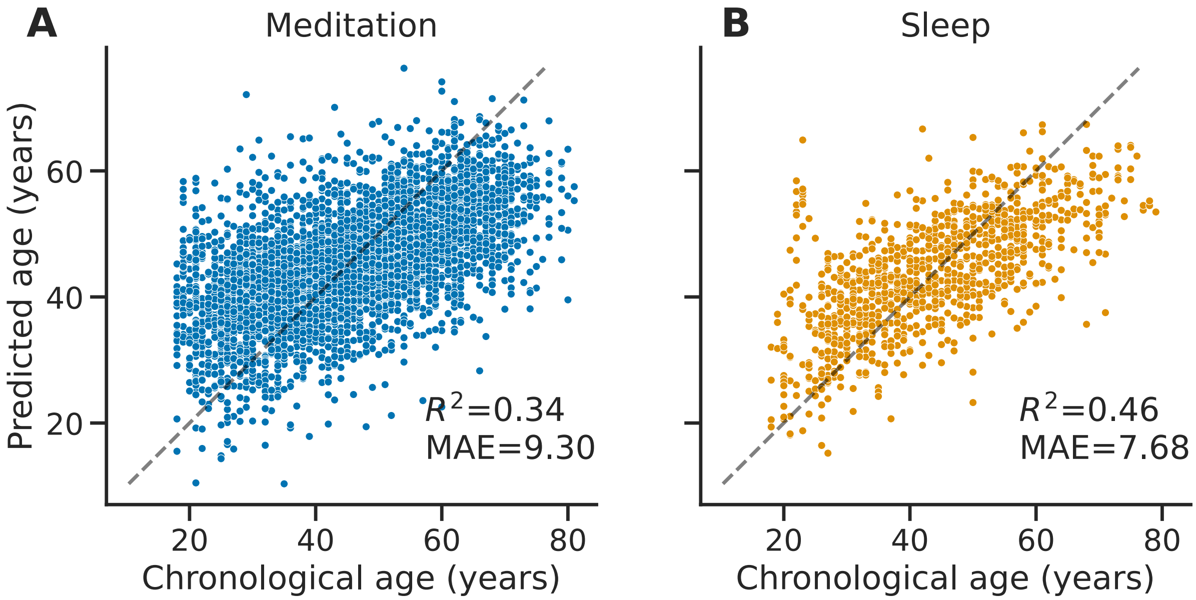

Figure S2: Scatter plots of chronological age versus predicted brain age for (**A**) meditation recordings and (**B**) sleep recordings. Cross-validated age predictions were obtained using cross-spectro-spatial power models trained over MMD or AMUSeD, respectively, with 10-fold cross-validation. R^2 and MAE are reported for the plotted predictions. Each point represents a single subject and the identity lines indicate the behavior of a perfect model.

Finally, the trained model was deployed as-is on the sleep recordings of AMUSeD and AMUSeD-long to produce hypnograms, *i.e.*, sequences of overnight sleep stage predictions, with 30 s resolution.

D Additional brain age prediction results

Fig. S2 shows the relationship between the brain age predictions obtained on MMD and AMUSeD and the true chronological age of each subject. A single prediction per recording was obtained by retraining and evaluating cross-spectro-spatial models with a 10-fold cross-validation scheme.

Fig. S3 presents the performance of brain age models trained on AMUSeD using different parts of the night. Fig. S4 shows longitudinal brain age estimates for four additional subjects.

E EEG spectral characteristics in MMD

A visual assessment of the α peak characteristics of the data in MMD reveals that both the α peak frequency, *i.e.*, the frequency of maximum power in the α band, as well as the α power, vary across age groups (see Fig. S5). Therefore, these features are likely useful for distinguishing age groups in our meditation datasets.

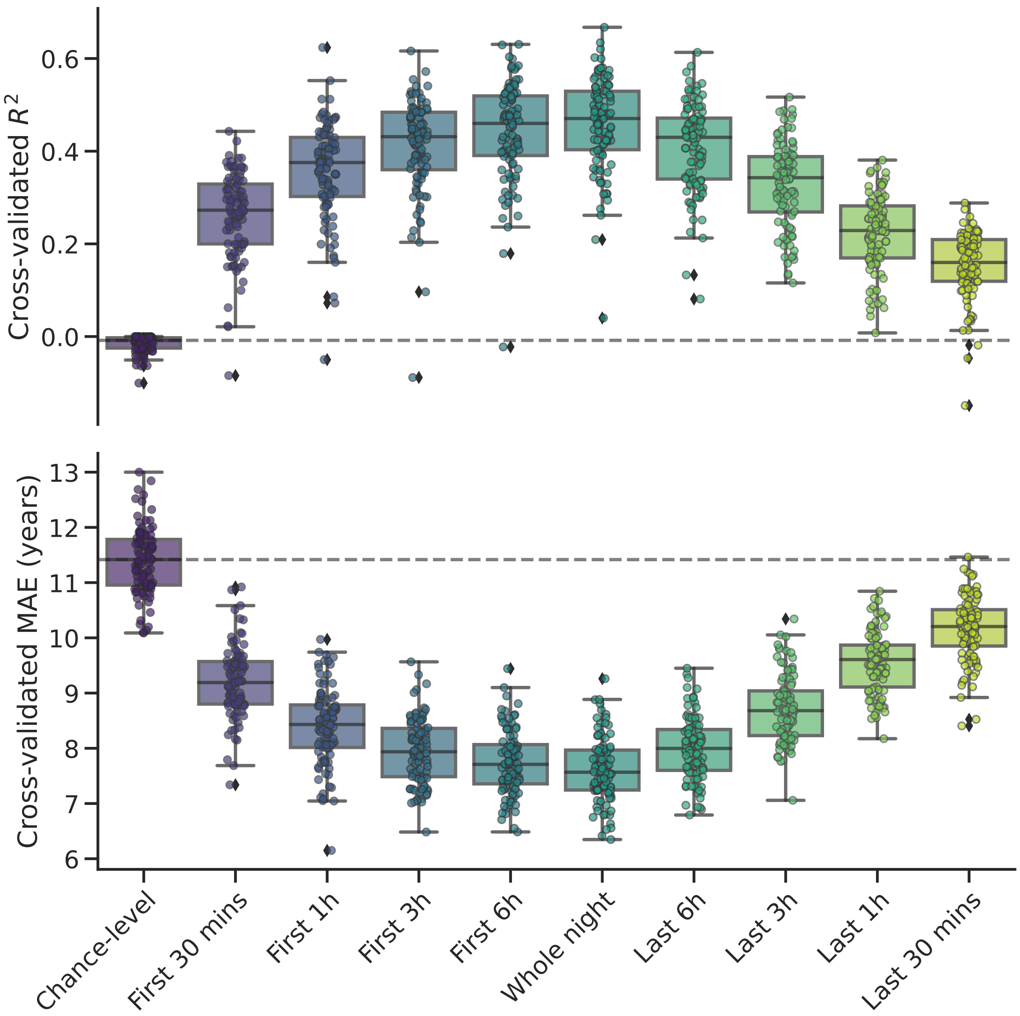

Figure S3: Cross-validated brain age prediction performance of models trained on different parts of overnight sleep recordings (Monte Carlo, 100 iterations, 10% testing data) measured with (**A**) R^2 and (**B**) MAE. See Fig. 1 for details. Separate models were trained on the first part of the night (first 30 mins, 1h, 3h or 6h) or the last part of the night (last 6h, 3h, 1h or 30 mins). While a longer duration generally helped performance, models based on the first part of the night performed better than those based on the last part, likely because it contains higher proportion of NREM sleep.

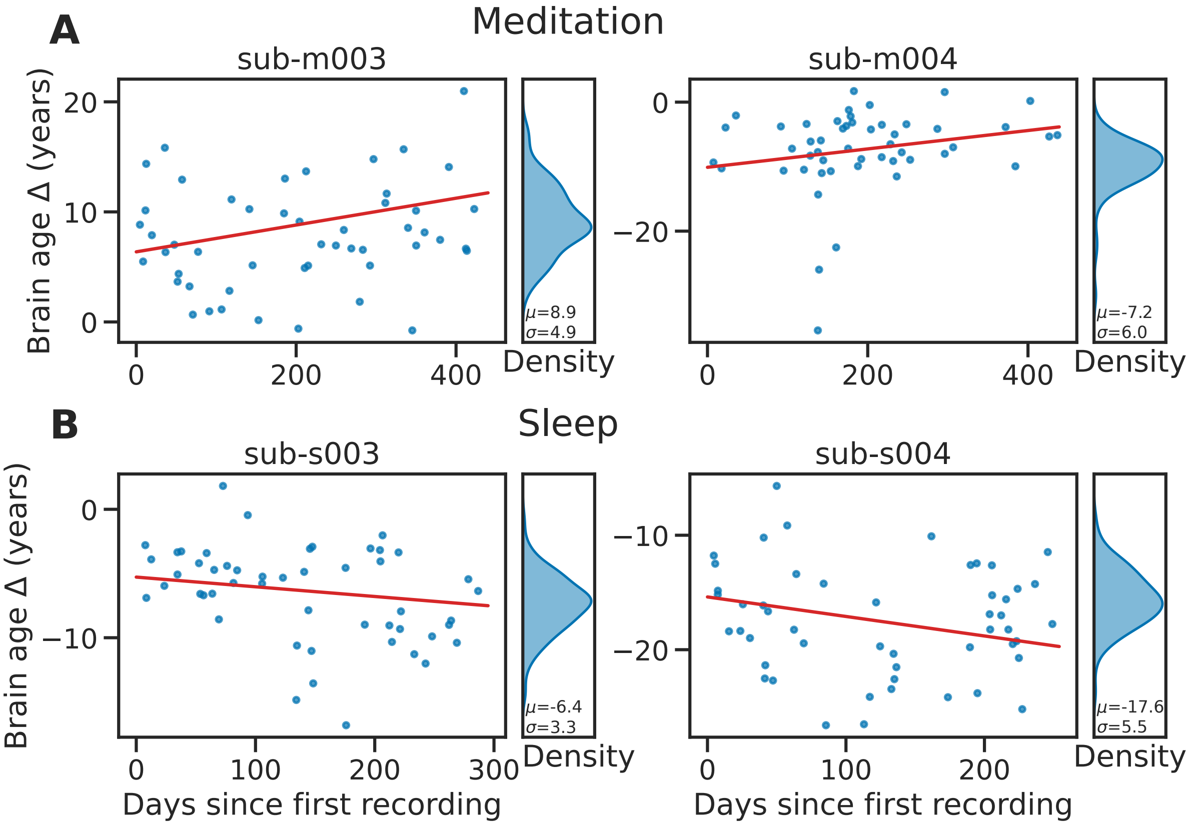

Figure S4: Longitudinal brain age Δ predictions for four additional subjects with multiple consecutive meditation (top) or sleep (bottom) recordings. See Fig. 4 for details.

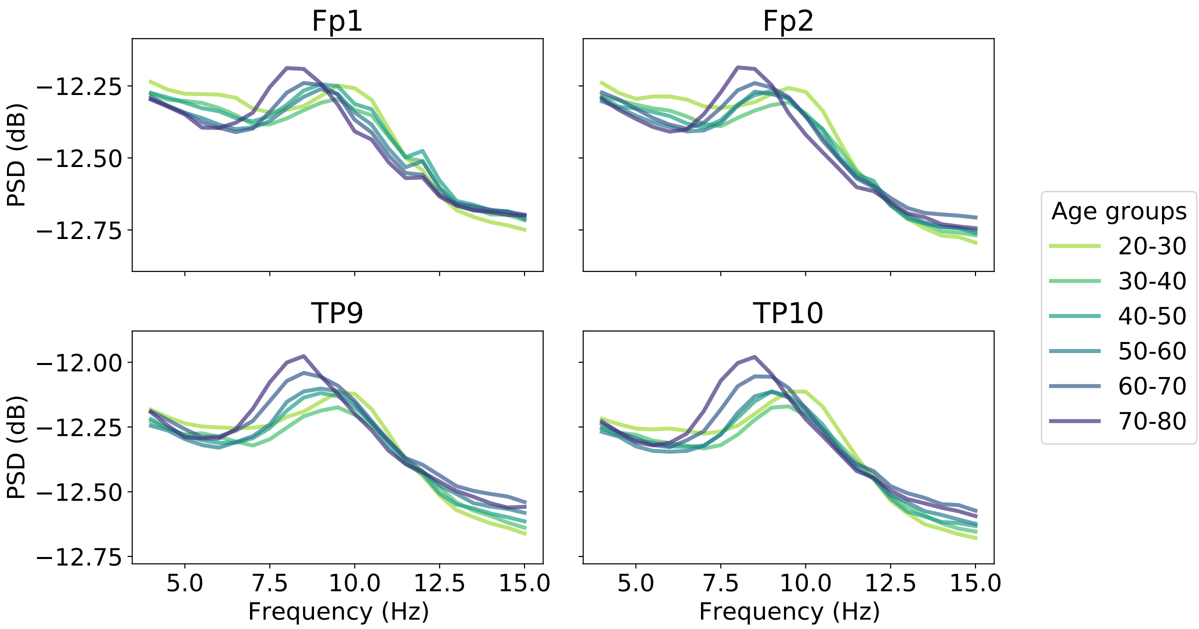

Figure S5: Visualization of the spectrum around the α band for recordings of MMD. Welch's periodogram with non-overlapping 4-s segments and median aggregation was applied to the 10-s windows preprocessed for use with filterbank models. The PSDs were then $\log_{10}$ −transformed and averaged inside each 10-year slice of the subjects. Older individuals tended to have a lower α peak frequency, as well as higher α peak power, in all four electrodes.

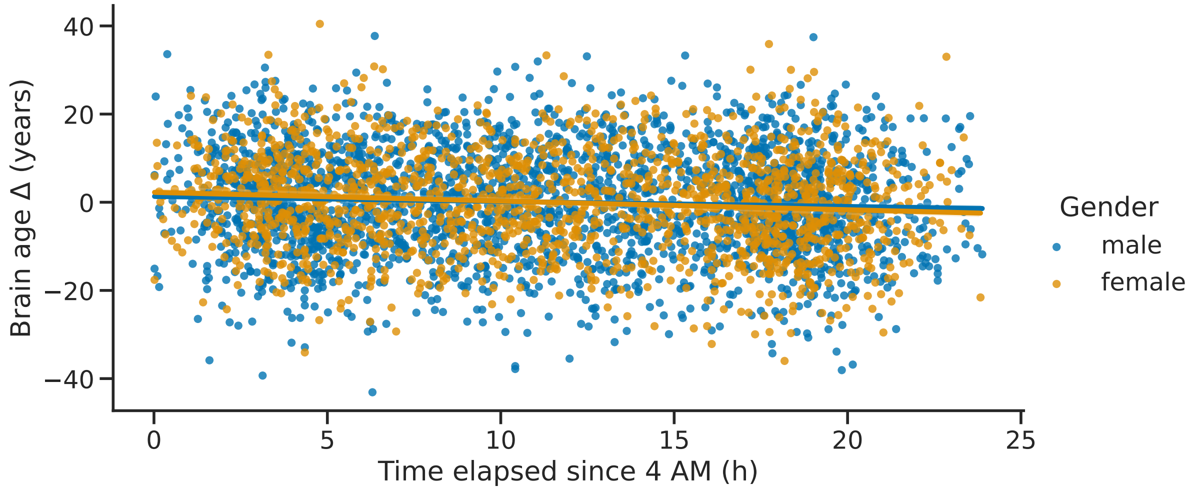

Figure S6: Effects of adjusted time of day (time elapsed since 4 AM) and gender on meditation-based brain age Δ. The cross-validated age predictions obtained on MMD are shown as a function of the time at which recordings were started. Subject gender is color-coded (blue for male, yellow for female). Linear fits display the gender-specific impact of adjusted time of day on the brain age metric.

F Time of day effects on brain age Δ

Fig. S6 shows brain age Δ measures for all recordings of the MMD dataset as a function of time of day. Brain age Δ and time of day are significantly correlated (Pearson's $\rho = -0.079$, $p < 10^{-5}$).

References

Abou Jaoude, M., Ravi, A., Niu, J., Banville, H., Torres, N. F. & Aimone, C. (2023). Automated sleep staging on wearable EEG enables sleep analysis at scale. In *2023 11th International IEEE/EMBS Conference on Neural Engineering (NER)*, pages 1–4. IEEE.

Abou Jaoude, M., Sun, H., Pellerin, K. R., Pavlova, M., Sarkis, R. A., Cash, S. S., Westover, M. B. & Lam, A. D. (2020). Expert-level automated sleep staging of long-term scalp electroencephalography recordings using deep learning. *Sleep*, 43(11):zsaa112.

Banville, H., Wood, S. U., Aimone, C., Engemann, D.-A. & Gramfort, A. (2022). Robust learning from corrupted EEG with dynamic spatial filtering. *NeuroImage*, 251:118994.

Berry, R. B., Budhiraja, R., Gottlieb, D. J., Gozal, D., Iber, C., Kapur, V. K., Marcus, C. L., Mehra, R., Parthasarathy, S., Quan, S. F., Redline, S., Strohl, K. P., Davidson Ward, S. L. & Tangredi, M. M. (2012). Rules for scoring respiratory events in sleep: update of the 2007 AASM manual for the scoring of sleep and associated events: deliberations of the sleep apnea

definitions task force of the American Academy of Sleep Medicine. *Journal of clinical sleep medicine*, 8(5):597–619.
